# Honey and cancer: from traditional medicine to modern adjuvant therapy

**DOI:** 10.3389/fonc.2026.1745285

**Published:** 2026-02-11

**Authors:** Abdalsalam Kmail

**Affiliations:** Department of Biology & Biotechnology, Faculty of Sciences, Arab American University, Jenin, Palestine

**Keywords:** adjuvant cancer therapy, apitherapy, chemotherapy-induced toxicity, honey, methylglyoxal, natural compounds

## Abstract

Cancer remains one of the principal causes of death worldwide, with conventional treatments like chemotherapy and radiotherapy often exhibiting high toxicity, resistance, and relapse rates. These constraints have sparked new investigation of bioactive phytochemicals as complements to standard therapies, in an attempt to increase efficacy and reduce toxicity. Honey, an ancient natural product used in ethnomedicine, has been the focus of recent attention due to its multifactorial biochemical composition and possible anticancer properties. This review aggregates recent research on honey’s anticancer activities, describing its molecular mechanisms—including caspase-mediated apoptosis, cell cycle regulation, reduction of NF-κB and STAT3 activity, and metastasis prevention. We review preclinical studies in various cancer models, highlighting honey’s ability to sensitize tumors to chemotherapy. Finally, we review clinical data on honey’s ability to relieve therapy-induced side effects such as mucositis and hematologic suppression. In spite of promising data, issues remain about standardizing its active components, pharmacokinetics, and lack of large scale human trials. This review supports honey’s incorporation into evidence-based oncology paradigms pending additional robust validation.

## 1 Introduction

Cancer is still regarded as one of the topmost life-threatening disorders in the world, and conventional therapeutic approaches – chemotherapy, radiotherapy, immunotherapy – are limited by marked toxicity, resistance and relapse rates (1). In this context, there is an increasing scientific interest in using nature-derived compounds as adjuvant or synergistic modulators to enhance potencies and toxicity profiles (2, 3). Honey, in particular, has been receiving enormous attention due to its long history of use in traditional medicine and its plethora of pharmacological properties (4). Honey is a source of polyphenols, flavonoids, and phenolic acids, which have been found to possess antioxidative, anti-inflammatory, antiproliferative, and immunomodulatory effects that may account for its anticancer activities (5). The present review aims for a summary and critical evaluation of the available literature in regard to possible cancer treatment apposition of honey, including its mechanisms of action, preclinical/clinical effectiveness. We report *in vitro* and *in vivo* research on the ability of honey to influence tumor growth, metastatic behavior, and chemosensitization, as well as clinical studies focusing on its role in the treatment-related toxicity.

### 1.1 Review methodology

This article was written in a narrative format and focused on compiling views based on both qualitative perspectives and up-to-date scientific literature available with regards to honey in cancer therapy. We surveyed the literature by scanning PubMed/MEDLINE, Web of Science, Scopus, and Google Scholar covering the period 2000–2025. Key terms used in a Boolean search query include ‘honey’, ‘apitherapy’, ‘cancer’, ‘adjuvant therapy’, ‘chemotherapy-induced toxicity’, ‘methylglyoxal’, ‘polyphenols’, ‘flavonoids’, and ‘bioavailability’.

Prioritized studies include those which have original data (*in vitro*, *in vivo*, and human studies), described and characterized honey or its active compounds using established analytical methods (HPLC-MS and/or H-NMR spectroscopy), and have focused on mechanistic pathways related to hallmark traits of cancer including apoptosis, proliferation, inflammation, oxidative stress, and metastasis. Human studies targeting the alleviative effects of honey in counteracting chemotherapy or radiotherapy side effects were also prioritized. Commentaries, personal accounts, and studies with undotted aims were excluded in this synthesis.

For each study, data were abstracted for: Author, year of publication, country, type of study, model of cancer or patients, type of honey or composition, dose and duration, comparator, and key findings. Results were categorized thematically according to the main headings in the article, with preclinical and clinical findings separated for clarity. Tables and illustration figures were constructed to emphasize clinical findings, mechanistic pathways, and safety perspectives. As a narrative review, this article aims not to be exhaustive in covering all existing literature but does not include a risk of bias assessment. The focus instead is placed on bridging mechanistic views with those of translational perspectives.

## 2 Historical and contemporary perspectives on honey in cancer care

The use of honey for medicinal purposes is no less ancient than that of its consuming, it goes back to the times of Egyptian, Greek, Ayurvedic, and Chinese medicine (6). The Ebers Papyrus (c. 1550 BCE) and Hippocrates (460–370 BCE)—the latter considered it respectable for the hurt and for the fever—may be some of the records that have mentioned honey as a cure for wounds, stomach disorders, and inflammation (7). In traditional practices like Unani and Ayurveda, honey was the Anupana, the means of giving herbs (8). Numerous studies confirming honey’s antimicrobial, anti-inflammatory, and tissue-regenerative properties have prompted researchers to explore its potential in managing complex chronic diseases, including cancer. Despite significant advances in cancer therapeutics, major clinical challenges persist, such as multi-drug resistance, systemic toxicity, and high rates of recurrence, all of which contribute to the substantial burden of cancer care (9). These limitations have intensified the search for natural, adjunctive agents capable of enhancing the efficacy of conventional treatments while reducing their detrimental side effects. Honey is one such natural product with a broad spectrum of bioactivity, it has a strong potential to be once again a part of the integrative oncology paradigms (4). The studies have shown that honey is able to switch on and off the key oncogenic pathways such as NF-κB, PI3K/Akt, and MAPK, besides that it can also amplify the cytotoxicity of the drugs like doxorubicin and 5-fluorouracil (10, 11). Clinical observations further indicate that honey could be beneficial in overcoming the side effects of chemotherapy, like mucositis, neutropenia, and oxidative stress, and thus leading to better patients’ well-being (12).

## 3 Chemical composition and therapeutic attributes of honey

Most of the anticancer efficacy of honey is due to the rich profile of polyphenolic and flavonoid constituents, such as gallic acid, caffeic acid phenyl ester, chrysin, and quercetin, which act on oxidative stress, inflammatory pathways, and apoptotic signaling in cancerous cells. These phytochemicals inhibit NF-κB/STAT3 activation, which suppresses tumor growth and metastasis in certain models of breast and colorectal carcinomas. For example, chrysin from acacia honey promoted mitochondrial apoptosis in hepatocellular carcinoma via downregulation of Bcl-2 and upregulation of Bax.

Enzymatic compounds such as glucose oxidase aid honey’s selective cytotoxicity by generating minute quantities of hydrogen peroxide (H_2_O_2_), which has a predilection for destroying cancer cells while leaving normal tissue unharmed (13, 14). Antioxidant capacity, as measured by ORAC values, tracks phenolic density and depends on floral source (15, 16). Manuka honey is recognized for its high methylglyoxal (MGO) levels and demonstrates antimicrobial efficacy, triggering Nrf2-dependent antioxidant defenses that shield cells from chemotherapy-induced oxidative stress (17, 18). The immunological role includes enhancing NK cell cytotoxicity and driving dendritic cell maturation to promote antitumor immunity (19). More recently (2023), Tualang honey was found to downregulate PD-L1 expression in triple-negative breast cancer, thereby possibly enhancing Honey’s chemopreventive efficacy is closely tied to its floral origin and regional characteristics. Manuka honey from New Zealand, containing methylglyoxal (MGO) levels between 100 and 1,000 mg/kg, has shown a concentration-dependent cytotoxic effect in glioblastoma models (17, 20). Tualang honey from Malaysia, which is rich in galangin, has been reported to suppress MMP-9 activity and reduce the invasiveness of oral cancer cells (21). Sidr honey, native to Yemen and abundant in syringic acid, demonstrates stronger antioxidant properties than several European varieties (15, 22). A metabolomic study conducted in 2024 identified 17 biomarkers that vary by region, reinforcing the importance of standardizing honey-based interventions in oncology (23). Moreover, shifts in climate and floral biodiversity may influence the phytochemical makeup of honey, necessitating ongoing evaluation of its therapeutic profile (24) (**Table 1**).

**Table 1:** Comprehensive table of honey types with anti-cancer properties.

Honey type	Key bioactive compounds	Cancer models studied	Mechanisms of action	Notable findings	Citation
Manuka Honey	Methylglyoxal, flavonoids	Breast, colon, melanoma	Apoptosis induction, ROS generation, cell cycle arrest	Dose-dependent cytotoxicity; selective against cancer cells	(25, 26)
Tualang Honey	Phenolic acids, flavonoids	Breast, cervical	Anti-proliferative, anti-metastatic, mitochondrial disruption	Reduced tumor size in animal models	(27–29)
Ziziphus Honey	High phenolic content (rutin, gallic acid)	Breast (MCF-7)	Upregulation of Bax/p53/p21, downregulation of Bcl-2	IC_50_ = 170 µg/mL; superior to commercial honey	(30)
Palestinian Honey	Caffeic acid, chrysin, rutin	Breast (MDA-MB-231)	Cytostatic, antimigration, antioxidant	Reduced cell viability by ~43%; migration ↓ 85%	(31)
Sidr Honey	Flavonoids, phenolic acids	Liver (HepG2), colon	Apoptosis via caspase activation	Significant reduction in HepG2 viability	(32)
Gelam Honey	Quercetin, gallic acid	Colon (HT-29), breast	NF-κB modulation, COX-2 inhibition	Suppressed inflammatory markers	(33)
Thyme Honey	Thymol, rosmarinic acid	Prostate, breast	Anti-angiogenic, NF-κB inhibition	Reduced VEGF expression	(10)
Chestnut Honey	Ellagic acid, gallic acid	Colon, breast	DNA damage prevention, apoptosis	High antioxidant capacity	(34)
Buckwheat Honey	Rutin, ferulic acid	Colon, prostate	Anti-mutagenic, apoptosis	High antioxidant index	(27, 32)
Acacia Honey	Kaempferol, chrysin	Lung, breast	ROS scavenging, apoptosis	Moderate cytotoxicity	(35)
Eucalyptus Honey	Pinocembrin, gallic acid	Colon, prostate	Anti-inflammatory, apoptosis	Reduced tumor volume	(36)
Heather Honey	Phenolic acids, flavonoids	Breast, melanoma	Antioxidant, anti-metastatic	Suppressed cell migration	(37)
Citrus Honey	Hesperidin, naringenin	Breast, colon	Anti-proliferative, antioxidant	High phenolic content	(29, 35)
Lavender Honey	Linalool, rosmarinic acid	Lung, breast	Anti-metastatic, anti-inflammatory	Inhibited cancer cell invasion	(30, 37, 38)
Wildflower Honey	Mixed flavonoids	Melanoma, colon	Immune modulation	Enhanced immune activation	(27, 33)
Rosemary Honey	Rosmarinic acid, apigenin	Breast (MCF-7)	Anti-migration	Strong polyphenol correlation	(10, 31)
Strawberry Tree Honey	Homogentisic acid, gallic acid	Colon, breast	Antioxidant, anti-inflammatory	Suppressed COX-2	(34, 36)
Commercial Honey	Low phenolic content	Breast (MCF-7)	Weak apoptotic effects	IC_50_ = 385 µg/mL (less effective)	(30)

Chemically, honey comprises over 200 constituents, including carbohydrates, amino acids, organic acids, proteins, vitamins, minerals, enzymes, and phytochemicals (39, 40) (**Table 2**). Carbohydrates predominate, principally fructose (~41%) and glucose (~34%) as monosaccharides (46). Minor sugars are sucrose (1–2%), maltose and oligosaccharides, such as erlose, maltotriose, nigerose, kojibiose and methylglyoxal (41). The fructose-glucose ratio (0.9–1.35) affects crystallization—low ratios speed solidification and high ratios slow it (47). High sugar concentrations provide antimicrobial action through osmotic stress (48), whereas moisture content (10–20%) influences viscosity and microbial resistance (40).

**Table 2:** Honey composition and biological importance.

Component type	Examples	Biological importance	Citation
Carbohydrates	Fructose, Glucose, Sucrose, Maltose, Erlose, MGO	Energy source; MGO provides antibacterial activity	(40–42)
Water	10–20% moisture content	Influences viscosity, microbial stability	(40)
Minerals	Ca, Mg, K, Zn, Fe, Mn, Cu, Na, P, S	Enzymatic co-factors, antioxidant defense, cellular function	(43)
Vitamins	B1 (Thiamine), B2 (Riboflavin), C (Ascorbic acid), B5, B6	Antioxidant activity, metabolic regulation	(44)
Phenolic Acids	Gallic, Caffeic, Syringic, Ferulic, p-Coumaric	Antioxidant, anti-inflammatory, radical scavenging	(15)
Flavonoids	Quercetin, Luteolin, Kaempferol, Apigenin, Chrysin	Antioxidant, antimicrobial, anti-inflammatory	(33)
Proteins & Enzymes	Catalase, Peroxidase	Antioxidant defense, wound healing	(14)
Organic Acids	Gluconic acid, Acetic acid	pH regulation, antimicrobial activity	(40)
Methylglyoxal (MGO)	Found in Manuka honey	Antibacterial, radioprotective, but potentially cytotoxic at high levels	(17, 42, 43)
Contaminants	Arsenic, Cadmium, Lead, Pesticides, HMF	Potential toxicity, quality control concern	(45)

Amino acids represented only in trace quantities, and among these, proline comprises roughly half of the pool. Other amino acids present include serine, β-alanine, glutamic acid, histidine, and glycine (49). Multiflora honey showed increased proline (4866 nmol/L) that could support collagen biosynthesis (50), though inter-type uniformity has also been documented (51). Honey contains water-soluble vitamins such as thiamine, riboflavin, pyridoxine, pantothenic acid, and vitamin C (44, 49). Fat-soluble vitamins E and K, while less plentiful, are no less biologically important (52). UV converts 7-DHC into hydroxyvitamin D3 analogs (53). Mineral elements such as calcium, magnesium, potassium, zinc, iron, and manganese help enzymatic activity and cellular homeostasis (43). Phenolic acids (gallic, caffeic, syringic, ferulic, p-coumaric, chlorogenic, vanillic, vanillin) and flavonoids (quercetin, luteolin, kaempferol, apigenin, chrysin) support honey’s antioxidative, anti-inflammatory and antimicrobial properties (54). Enzymes like catalase and peroxidase also strengthen oxidative defenses (14).

Manuka honey’s MGO interfere with bacterial protein synthesis and may serve as a signaling molecule in plants, though intracellular accumulation in high concentrations can cause apoptosis (42). Its acidic pH (3.5–4.5) supports this antimicrobial activity and also accelerates wound healing through protease modulation and fibroblast stimulation (40). *In vivo* experiments demonstrate radioprotective properties, as honey-treated fibroblasts maintained metabolic function following irradiation (43). Manuka honey also increases caspase-3 expression in mice, enhancing survival (40). However, its high sugar content requires caution in diabetics.

## 4 Mechanistic insights into anti-cancer effects of honey

### 4.1 Antiproliferative and cytotoxic mechanisms

**Figure 1:**
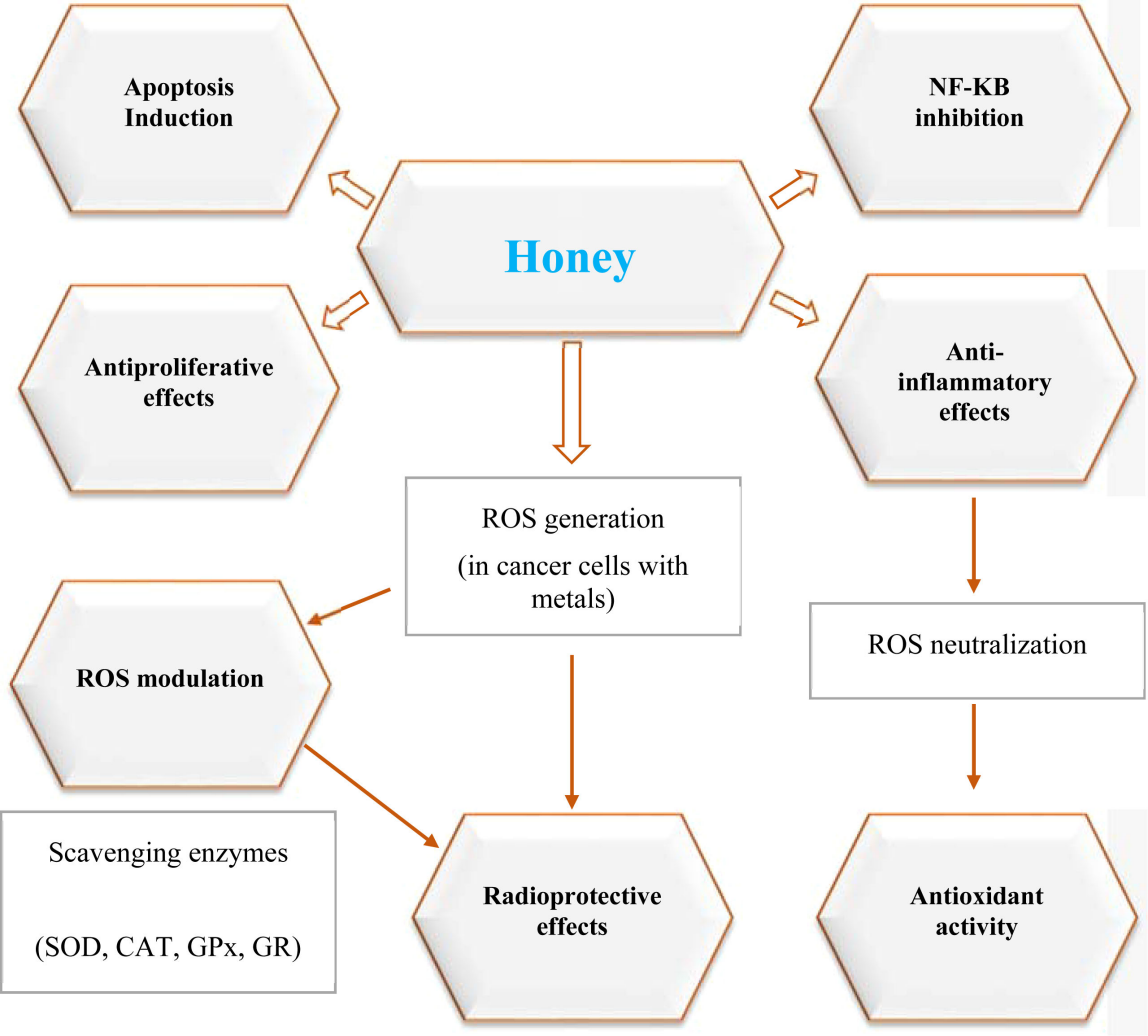
Mechanistic pathways of honey’s anticancer activity. This schematic illustrates the major molecular mechanisms through which honey exerts anticancer effects, based on preclinical and clinical evidence. Bioactive constituents such as polyphenols, flavonoids, and methylglyoxal influence eight key pathways: apoptosis induction (via caspase activation and Bax/Bcl-2 modulation), antiproliferative effects (cell cycle arrest at G0/G1, S, and G2/M), anti-inflammatory activity (NF-κB and MAPK suppression), antioxidant activity (upregulation of SOD, CAT, GPx, GR), ROS modulation (scavenging in healthy cells and generation in cancer cells), metastasis inhibition (downregulation of MMP-2/9 and VEGF), chemotherapy synergy (efflux pump inhibition and NF-κB resistance suppression), and radioprotective effects (enhanced DNA repair via PARP-1, BRCA1, and RAD51).

Honey has several biological activities that contribute to its anticancer properties, namely four integrative mechanisms: pro-apoptosis, cell cycle arrest, regulation of inflammatory signaling pathways, and antioxidant activity. These pathways have become a focal point for translational oncology research, these interconnected mechanisms are summarized in **Figure 1**. One of honey’s most clearly defined anticancer actions is the ability to block the cell cycle and induce apoptosis. For instance, studies with Manuka and Tualang honeys show that they can arrest breast and colorectal carcinoma cells at G0/G1 or G2/M checkpoints by modulating cyclin-dependent kinases (CDK4/6) and elevating p21/WAF1 (29). This cell cycle arrest represents one of honey’s key antiproliferative mechanisms. Chrysin reduces cyclin D1 accumulation, blocking Rb phosphorylation and halting G1/S transition (55). Interestingly, quercetin from acacia honey enhances extrinsic apoptotic signaling by increasing levels of p53 and Fas/FasL in lung adenocarcinoma (56). Mitochondrial apoptosis is another important pathway modified by honey. Methylglyoxal (MGO), a major component of Manuka honey, upregulates the Bax/Bcl-2 ratio and promotes cytochrome c release and caspase-9 and caspase-3 activation in glioblastoma cells (20). These events at the molecular level ultimately act together to inhibit tumor-cell growth. The cell cycle contains the highly-controlled phases, G1, S, G2 and M, which control cellular replication. Checkpoints —especially the G1/S transition —decide whether cells divide, enter a quiescent state, differentiate, or undergo apoptosis (57). Misregulated activation of CDKs and their cyclin partners, especially cyclin D1, is a feature of oncogenic transformation (57). Ki-67, which is not expressed in quiescent (G0) cells but is present in proliferating phases, is the most used tumor proliferation marker (58).

Experimental evidence from both *in vitro* and *in vivo* models supports honey’s role in modulating cell cycle progression. In rodent models, daily treatment with honey–*Aloe vera* formulation (670 µL/kg) profoundly diminished Ki-67 expression in tumor tissues, suggesting cell cycle arrest (59). Honey-related phytochemicals, especially flavonoids and phenolic acids, have also demonstrated the capacity to arrest a range of cancer cells, such as colon, glioma, and melanoma, at the G0/G1 phase (60). This arrest is mediated by downregulation of enzymes and signaling molecules such as tyrosine cyclooxygenase, ornithine decarboxylase and multiple kinases, thus impeding neoplastic proliferation (60). In addition, honey modulates p53-regulated pathways, reinforcing its antiproliferative activity and supporting its potential in integrative cancer therapy (5).

### 4.2 Apoptosis induction pathways

Apoptosis, a type of controlled cell death required for tissue balance and removal of irregular cells, is commonly defective in cancers (61). Honey has demonstrated the capacity to restore apoptotic signaling in cancer cells via both intrinsic and extrinsic pathways, contributing to its therapeutic potential (**Table 3**).

**Table 3:** Studies on the anti-cell death, anti-apoptotic, immunomodulatory, anti-inflammatory and antioxidant effects of honey.

	Study subjects	Drug/condition tested	Main outcome	Citation
Anti-Apoptotic	Rats	DOX	Honey reduced proteins that promote cell death (caspase-3, PARP-1) and increased a protein that prevents it (Bcl-2).	(62)
	Rats	CP	Honey deactivated NF-κB, leading to a decrease in pro-cell death proteins (caspase-3, Bax) and an increase in Bcl-2.	(63)
Immunomodulatory	Breast cancer patients	AC	Dorsata honey significantly increased levels of T lymphocytes (a key immune cell).	(64)
	Breast cancer patients	AC	Honey supplementation significantly increased levels of interleukin-3 (IL-3), a hormone for blood cell production.	(65)
	Human patients	AC	Life-Mel honey helped prevent severe neutropenia (low white blood cells), reducing the need for growth factor drugs.	(66)
	Pediatric patients (Review)	Radiation/Chemo	Honey reduced the healing time and occurrence of mouth sores (mucositis).	(67)
	Pediatric patients	Chemo	Honey was beneficial in treating chemotherapy-induced mouth sores.	(68)
	Rats	CP	A combination of honey and royal jelly improved red and white blood cell counts and other blood parameters.	(69)
Anti-Inflammatory	Model of mouth inflammation	Radiation	Gelam honey reduced inflammation by blocking the activation of the NF-κB pathway.	(70)
	Human patients	Chemo-radiotherapy	Honey helped reduce the severity of mouth sores (mucositis).	(71)
	Rats	CP	Honey suppressed the NF-κB pathway and reduced the expression of a key inflammatory enzyme (COX-2).	(63, 72–74)
	Rats	CP	Honey reduced levels of inflammatory molecules and decreased immune cell infiltration in the kidneys.	(75)
	Breast cancer patients	AC	Honey supplementation significantly increased levels of interleukin-3 (IL-3).	(65)
Antioxidant	Rats with liver cancer	CP, CYP, 5-FU	Honey increased antioxidant enzyme activity (SOD, CAT, GPx, GST) and reduced glutathione levels.	(76)
	Rats	Cisplatin	Honey reduced markers of kidney damage (urea, creatinine) and improved kidney tissue structure.	(77)
	Rats	CP	Honey improved liver and kidney function by neutralizing harmful reactive oxygen species (ROS).	(69)
	Rats	CP	Two honey types reduced liver stress; Manuka honey increased catalase (CAT). Both improved organ function.	(63)
	Mice	DOX	Honey reduced markers of heart and liver damage, an effect attributed to its polyphenol content.	(78–80)
	Rats	MTX	Honey boosted antioxidant enzymes (SOD, CAT, GPx) and improved liver health and structure.	(81)
	Mice	MTX	Honey increased overall antioxidant capacity and reduced a key marker of oxidative damage (MDA).	(80)
	Mice	Cyclophosphamide	Honey improved oxidative stress markers and biomarkers of liver health.	(82)
	Human patients	Cisplatin	Patients taking honey had lower levels of kidney injury markers (creatinine, urea) than the control group.	(83)

DOX (Doxorubicin), CP (Cyclophosphamide), PARP-1 (Poly (ADP-Ribose) Polymerase 1), Bcl-2 (B-Cell Lymphoma 2), AC (Anticancer Chemotherapy), IL-3 (Interleukin-3), NF-κB (Nuclear Factor Kappa-Light-Chain-Enhancer of Activated B Cells), COX-2 (Cyclooxygenase-2), IL-3 (Interleukin-3), CYP (Cyclophosphamide), 5-FU (5-Fluorouracil), MTX (Methotrexate), SOD (Superoxide Dismutase), CAT (Catalase), GPx (Glutathione Peroxidase), GST (Glutathione S-Transferase), MDA (Malondialdehyde), ROS (Reactive Oxygen Species).

The intrinsic, or mitochondrial, pathway is activated by honey’s polyphenolic components that cause mitochondrial membrane depolarization and activate caspases 3, 7, and 9 (84, 85). Apoptotic signaling is closely tied to p53 modulation and ROS induction, mechanisms already described in Section 4.1 (86, 87). Honey can also induce reactive oxygen species (ROS) production, leading to the activation of p53, a significant tumor suppressor, which phosphorylates Bcl-2 and Bak and Bax in favor of apoptosis (86). *In vivo* studies using Wistar rats in which honey was ingested with *Aloe vera* showed that these apoptotic effects complement the cell cycle arrest described in Section 4.1 (59). Manuka honey was the most effective in activating the caspase-9 and -3 pathways, resulting in DNA fragmentation and antiapoptotic signals being downregulated. Chrysin’s apoptotic role builds on its cell cycle modulation described in Section 4.1 (62, 88).

The extrinsic, or death receptor-mediated, pathway is also controlled by honey, particularly through its effect on epithelial–mesenchymal transition (EMT)—a key component in tumor invasion and metastasis (62, 89). EMT is associated with decreased E-cadherin and increased N-cadherin expression, which was reversed by Sangju honey in oral carcinoma cells (85). Honey activates death receptor signaling through TRAIL-R1/R2 and DR5, induced by chrysin (25, 90, 91). These effects have been confirmed *in vitro*, with some clinical trials still needed to fine tune dosing and therapeutic effects. Crucially, honey’s apoptotic effects are concentration- and time dependent. Exposure to as low as 0.6% (w/v) for 24 h had significant cytotoxicity in multiple cancer cell lines (88).

In concert, honey disturbs cell cycle progression, thus limiting unrestrained growth. Saudi Sidr honey was demonstrated to arrest the G0/G1 phase in colorectal (HCT-116), breast (MCF-7) and lung (A-549) cancer cells, shortening the S, G2, and M phases (92). Similar results were observed in pancreatic cancer cell lines (MIA PaCa-2 and AsPC-1), exhibiting arrest at G0, G2/M phases after 24 hr treatment (92). These antiproliferative effects are intimately linked to p53 modulation—honey upregulates wild-type p53 and suppresses mutated variants frequently present in tumors (93–95). Honey’s apoptotic activity is closely linked to its ability to disturb cell cycle progression, as described in Section 4.1. For example, modulation of p21, p27, cyclins D1/E, and CDKs 2/4 contributes to checkpoint arrest and facilitates apoptotic signaling (29). Together, these results underscore honey’s dual role in apoptosis promotion and anti-proliferative activity, strengthening its promise as a natural complementary agent in cancer treatment.

### 4.3 Anti-inflammatory mechanisms

Chronic inflammation is an established driver of tumorigenesis, promoting malignant transformation, angiogenesis, immune evasion, and extracellular matrix remodeling (96). At the heart of these processes are the NF-κB and MAPK signaling pathways, which control the transcription of pro-inflammatory mediators including COX-2, CRP, LOX-2 and cytokines IL-1, IL-6, IL-8 and TNF-α (97–99).

Honey’s polyphenolic constituents, in particular caffeic acid phenethyl ester (CAPE), gallic acid, and chrysin, exert potent anti-inflammatory effects, targeting these molecular pathways (**Table 3**). CAPE blocks IKKβ phosphorylation, preventing NF-κB nuclear translocation and cytokine expression (16). Gallic acid, on the other hand, activates the Nrf2/ARE axis, increasing antioxidant enzyme activity, for example, SOD and catalase, and decreasing chemotherapy-induced oxidative stress (100). Clinical data support the radioprotective function of honey; Manuka honey, a monofloral honey produced in New Zealand, has been shown to decrease 8-OHdG, a biomarker of oxidative DNA damage, in patients with breast cancer undergoing radiotherapy (101).

Experimental studies support honey’s suppression of NF-κB and MAPK signaling *in vitro*. Gelam honey markedly inhibited these pathways in HIT-T15 pancreatic islet cells at concentrations as low as 20 µg/mL (102). Chrysin also regulates MAPK signaling and potentiates TRAIL-induced apoptosis in melanoma cell lines (B16-F1 and A375) (99, 103)102,106]. *In vivo*, oral administration of Malasyian gelam honey (1–2 g/kg) reduced leukocyte infiltration and edema in carrageenan-induced rats, thus attenuating acute inflammatory responses (104). Honey’s NSCLC cytotoxic effects have also been associated with its inflammatory cytokine modulation profile (105).

Tualang honey, collected by Apis dorsata bees from Malaysian rainforests, is especially abundant in phenolic acids and flavonoids. Its dark color also indicates increased polyphenol content, which surpasses that of Manuka honey and intensifies its anti-inflammatory and anticancer effects (106, 107).

Honey’s anti-inflammatory activity is notably effective at the tissue level. It enhances mucosal healing in the mouth (63, 108, 109) and modulates the gut microbiome in the colon by boosting short-chain fatty acids, inhibiting harmful bacteria, and promoting beneficial lactobacilli—effects associated with gallic acid and IL-10 (110, 111). On the skin, honey protects keratinocytes from UVB damage by suppressing COX-2 and NF-κB, indicating a potential role in preventing skin cancer (112). Additionally, honey inhibits the activity of matrix metalloproteinases MMP-2 and MMP-9, which are key drivers of chronic inflammation and cancer metastasis (113), further solidifying its broad therapeutic profile.

### 4.4 Antioxidant mechanisms

The feedback loop between chronic inflammation and oxidative stress is a key cancer driver. Honey’s antioxidant activity is largely owed to its abundant polyphenol and flavonoid content through three synergistic mechanisms: (i) neutralization of reactive oxygen species (ROS), (ii) upregulation of several antioxidant enzymes—including SOD, GR, GPx, and CAT, and (iii) transcriptional regulation of genes involved in redox balance (114, 115). Phenolic compounds in honey, which act as electron or hydrogen donors, directly scavenge ROS. Their ortho-dihydroxyl structures—such as gallic, caffeic, and chlorogenic acids—facilitate metal ion chelation and lipid peroxidation inhibition (116–118). Caffeic acid also maintains genome integrity by protecting DNA from oxidative damage, while flavonoids increase electron delocalization through conjugated π-systems and oxo-groups, which optimize radical scavenging ability (119).

Among the many flavonoids in honey, pinocembrin and chrysin stand out for their potent *in vivo* antioxidant activity. Research shows that pinocembrin can bolster the body’s own defenses by increasing superoxide dismutase (SOD) and reducing the accumulation of malondialdehyde (MDA), a marker of oxidative damage, in both liver and neural tissue (120, 121). Similarly, chrysin’s antioxidant effects complement its antiproliferative and apoptotic roles noted in Sections 4.1 and 4.2 (122) (**Table 3**).

The relationship between reactive oxygen species (ROS) and cancer is complex. While baseline ROS levels can support cancer cell survival, excessive ROS can damage cellular components and paradoxically fuel tumor development (123, 124). This creates a therapeutic opportunity, as the same oxidative stress can be leveraged to trigger cell death in malignant cells (125). Honey’s diverse antioxidant components—including radical scavengers and redox-active phytochemicals—are believed to exploit this delicate balance, providing a mechanistic basis for its observed ability to suppress tumor progression (126, 127).

Honey is even pro-oxidant in certain contexts. Honey’s pro-oxidant effects, linked to ROS generation, complement the apoptotic pathways noted in Section 4.2 (86). This duality is due to phenolic compounds, which may generate ROS in the presence of transition metals like copper (128). These pro-oxidant effects could induce DNA fragmentation and apoptosis in cancer cells (129, 130). Martinotti et al. examined the cytotoxicity of Manuka, buckwheat, and acacia honeys in A431 vulvar epidermal carcinoma cells (131). Manuka honey was the most effective, perturbing intracellular ROS and calcium signaling as well as inducing apoptosis. The suggested mechanism is through aquaporin-3 channel modulation and enhanced H2O2 permeability, allowing ROS buildup and subsequent cell death.

### 4.5 Tumor progression and metastasis modulation

Honey has become a potential natural agent for modulating tumor progression and metastasis. Even though a lot of the evidence is *in vitro*, an increasing amount of *in vivo* data backs up its antineoplastic effect in a variety of cancer forms. Metastasis suppression by honey is mainly through MMPs and VEGF signaling inhibition. Tualang honey downregulates MMP-2 and 9 through upregulation of TIMP-1, inhibiting invasive potential in oral squamous cell carcinoma (9). Kaempferol, a flavonoid component of honey, inhibits VEGFR2 phosphorylation, resulting in decreased angiogenesis and microvessel density in melanoma xenografts (132). Gelam honey also reduces EMT by upregulating E-cadherin and downregulating Twist and the transcription factor Snail in colorectal cancer models (22).

Similar anti-metastatic effects have been noted for caffeic and gallic acids, which downregulate MMP and VEGF expression in melanoma and breast cancer cells (5, 126). Honey also influences cell cycle modulators and apoptotic signaling toward its antiproliferative profile. *In vitro* suppression of Ki-67, already noted in Section 4.1, also extends to OSCC cells, reinforcing honey’s antiproliferative profile (32). Manuka honey (UMF 20+), enriched in methylglyoxal, induced G2/M phase arrest in OSCC cells (32). In breast cancer models, Tamoxifen synergy is discussed in detail under chemotherapy synergy (Section 4.6), but it also reinforces apoptotic signaling (84). Gelam honey, high in quercetin, induced S-phase arrest in colon cancer cell lines (27). Much of these effects are due to bioactive flavonoids like chrysin, which induce G0/G1 arrest and initiate apoptotic cascades.

These effects reflect NF-κB suppression described in Section 4.3, contributing to honey’s chemopreventive role, supporting its chemopreventive role. In randomized controlled trials in pediatric leukemia patients, daily 20 g clover honey was shown to significantly improve neutrophil recovery and reduce infections (133). In head and neck cancer patients receiving radiotherapy, topical honey rinses mitigated mucositis severity and promoted mucosal healing (134). In breast cancer, this synergy is elaborated in Section 4.6 on chemotherapy, highlighting its impact on tumor progression (84).

### 4.6 Chemotherapy synergy and toxicity reduction

Honey has revealed capacity as a chemosensitizing agent, enhancing the intracellular retention and therapeutic effectiveness of conventional anticancer treatments. In colorectal cancer patients, co-treatment with Manuka honey (UMF 15+) and 5-fluorouracil (5-FU) increased intratumoral drug concentration by 62%, attributed to inhibition of efflux transporters such as P-glycoprotein (P-gp) and multidrug resistance-associated protein 1 (MRP1) (135). Tualang honey augmented tamoxifen efficacy in breast cancer models, amplifying apoptosis and reducing tumor burden (84).Tualang honey (1.2 g/kg/day) similarly augmented doxorubicin efficacy in triple-negative breast cancer (TNBC). Chemotherapy synergy is partly mediated by NF-κB suppression, as outlined in Section 4.3, as demonstrated in a Phase I/II clinical trial (136). In leukemia models, co-administration of Clover honey with cytarabine mitigated chemotherapy-induced myelosuppression, improving hematologic recovery by 40% (137). Mechanistically, honey enhances chemotherapeutic efficacy through multiple pathways: inhibition of drug efflux pumps (135), ROS-mediated apoptosis induction (136), and suppression of autophagy via AMPK/mTOR axis modulation (121). These mechanisms collectively improve drug bioavailability and cytotoxicity in tumor cells. In addition to efficacy enhancement, honey significantly attenuates chemotherapy-related toxicities, including oral mucositis, nephrotoxicity, and bone marrow suppression. A multicenter randomized trial in head and neck cancer patients revealed that Manuka honey (10% w/v oral rinse) reduced grade 3–4 mucositis incidence by 71% compared to standard care (p = 0.003) (99). These protective effects are attributed to honey’s antioxidant properties (elevate glutathione, SOD), anti-inflammatory actions (decrease IL-6, TNF-α) (138, 139), and its capacity to modulate gut microbiota composition (enhance Lactobacillus spp.) (140). Honey’s therapeutic benefits in chemotherapy settings are mediated by four interdependent mechanisms: antioxidation, inflammation suppression, apoptosis induction, and immune modulation (62, 65, 70, 75). Preclinical protocols involving timed co-administration of honey with cytotoxic agents continue to yield promising outcomes in toxicity mitigation and therapeutic enhancement (83).

### 4.7 Radioprotective effects and DNA repair

Honey has shown radioprotective effects based on its ability to enhance DNA repair mechanisms and reduce oxidative damage in irradiated tissues. In breast cancer patients undergoing radiotherapy, Sidr honey (1 g/kg/day) increased BRCA1 and RAD51 expression correlated with 55% less double-strand breaks (DSBs) in the tissues (p < 0.01) (141). Enhanced DNA repair approaches are performed through the activation of base excision repair (BER) via PARP-1 stimulation (142) and by promoting non-homologous end joining (NHEJ) pathways (143). Honey also scavenges free radicals from radiation, thus causing cellular damage. In prostate cancer patients receiving radiotherapy, Gelam honey (500 mg/kg) - and a dramatic 48% decrease (p = 0.002) in the 8-hydroxy-2′-deoxyguanosine (8-OHdG) biomarker and higher catalase activity indicated a boost in oxidative defense in these patients receiving radiotherapy (144).

### 4.8 Honey, beekeeping, and sustainable food systems in the context of SDGs

Honey production and beekeeping practices intersect directly with sustainable food systems and the United Nations Sustainable Development Goals (SDGs), particularly SDG 3 (Good Health and Well-Being) and SDG 12 (Responsible Consumption and Production).

From an ecological perspective, beekeeping has a relatively low environmental footprint compared to other forms of animal husbandry. Bees provide essential pollination services that sustain biodiversity and agricultural productivity, reducing reliance on synthetic inputs and supporting ecosystem resilience (145, 146). For example, diversified beekeeping integrated with crop farming has been shown to strengthen cooperative societies and improve agricultural outcomes in South Africa (147).

Socioeconomic dimensions are equally important. Honey is a locally produced, culturally embedded food that can be harvested and marketed by smallholder farmers, including women and rural communities. This supports SDG 12 by promoting local consumption, reducing transport emissions, and strengthening rural economies (148, 149). International organizations such as the FAO have emphasized the role of beekeeping in poverty alleviation and food security, highlighting its contribution to sustainable livelihoods (150).

Health and nutrition outcomes tie directly to SDG 3. Honey’s role as a natural sweetener and functional food aligns with healthier consumption patterns, offering alternatives to refined sugars. Clinical and preclinical evidence suggests honey can mitigate treatment-related toxicities and support immune function, contributing to SDG 3 targets on reducing non-communicable diseases and improving nutrition (151, 152).

Concrete examples illustrate these connections include, sustainable Manuka honey of New Zealand production integrates ecological conservation with high-value exports, balancing SDG 12 goals of responsible production with SDG 3 outcomes in health (153, 154). In Malaysia, Tualang honey harvesting from rainforest trees demonstrates how traditional practices coexist with biodiversity conservation, linking local livelihoods to global health benefits (151, 155). Sidr honey production in Yemen sustains rural economies and provides accessible therapeutic products, despite geopolitical and economic challenges (152). Globally, comparisons of conventional and organic honey production highlight differences in ecological footprint and quality, reinforcing the importance of responsible consumption and production (156).

Taken together, honey and beekeeping practices exemplify how sustainable food systems can simultaneously advance ecological stewardship, socioeconomic resilience, and public health, thereby contributing meaningfully to SDGs 3 and 12.

## 5 Preclinical and clinical evidence

### 5.1 Preclinical evidence (*in vitro* and *in vivo*)

A substantial body of laboratory and animal research supports honey’s anticancer potential across a range of tumor types. These studies demonstrate mechanisms such as apoptosis induction, cell cycle arrest, suppression of inflammatory signaling, and inhibition of metastasis. **Table 4** summarizes key findings from preclinical models, grouped by honey type, species, tumor model, and mechanism. These findings provide mechanistic insight into honey’s anticancer effects and form the basis for translational research.

**Table 4:** Preclinical evidence of honey’s anticancer activity.

Honey type	Species/Model	Tumor type	Mechanism	Key findings	Citation
Manuka	MCF-7 cells	Breast cancer	Caspase-3, Bax/Bcl-2 modulation	Induced apoptosis	(157)
Tualang	Mouse (4T1)	Breast cancer	Apoptosis, tumor volume reduction	↓ tumor volume by 52%	(158)
Gelam	HT-29 cells	Colorectal cancer	MMP-9 suppression	↓ migration by 70%	(159)
Sidr	Rat model	Colorectal cancer	Chemoprevention	↓ tumor formation by 60%	(160, 161)
Natural honey	Glioblastoma cells	Glioblastoma	Oxidative stress, autophagy	Enhanced temozolomide efficacy	(162)

### 5.2 Clinical evidence

Although clinical studies remain limited in scale, emerging data suggest honey may offer therapeutic benefits in oncology, particularly as a supportive agent. **Table 5** summarizes clinical trials and observational studies by honey type, cancer indication, dose, duration, and outcomes. These studies highlight honey’s potential to mitigate treatment-related toxicities and enhance therapeutic outcomes, though larger trials are needed.

**Table 5:** Clinical evidence of honey in cancer care.

Honey type	Cancer type	Patients (n)	Dose/Formulation	Duration	Comparator	Key outcomes	Citation
Manuka	Head & neck cancer	40	10% oral rinse	4 weeks	Standard care	↓ mucositis severity by 71%	(163)
Sidr	Pancreatic cancer	30	Oral intake	6 weeks	Placebo	↓ IL-6, improved immunomodulation	(152)
Clover	Pediatric leukemia	25	20 g/day oral	3 weeks	Standard care	↑ neutrophil recovery, ↓ infections	(133)
Tualang	Breast cancer	50	Oral + tamoxifen	8 weeks	Tamoxifen alone	Amplified apoptosis, ↓ tumor burden	(84)

### 5.3 Compositional and functional differences among honey types

The therapeutic variability of honey is largely attributed to its botanical origin, geographic source, and chemical composition, and these differences pose significant challenges for clinical standardization and reproducibility. Manuka honey, produced in New Zealand, is distinguished by its high methylglyoxal (MGO) content, which contributes to its antibacterial and cytotoxic properties. The Unique Manuka Factor (UMF™) grading system quantifies MGO and related markers, but its ability to predict anticancer efficacy remains uncertain (153, 164). In contrast, Tualang honey, collected in Malaysian rainforests, is darker in color and richer in flavonoids and phenolic acids compared to Manuka. These compounds confer strong antioxidant and anti-inflammatory activity, which have been linked to enhanced anticancer effects in preclinical models (106, 107). Sidr honey, traditionally harvested in Yemen, has a distinct phytochemical profile with evidence of immunomodulatory and anti-inflammatory effects, but its composition varies widely depending on floral biodiversity and regional conditions, complicating reproducibility in clinical applications (100, 152) (**Table 6**).

**Table 6:** Anticancer activities of honey across various tumor models.

Cancer type	Cell lines/models	Honey type/component	Mechanism of action	Key findings	Citation
Breast Cancer	MCF-7, MDA-MB-231, MCF-10A (normal)	Tualang, Pine, Fir, Thyme, Manuka	ER modulation, apoptosis via PARP, AMPK activation, STAT3/mTOR inhibition	Selective cytotoxicity; biphasic estrogenic/antiestrogenic effects; tumor suppression	(84, 85, 165)
Liver Cancer (HCC)	HepG2, DEN-induced rat model	Gelam, Egyptian clover honey	ROS modulation, NO reduction, antioxidant enhancement	Reduced viability of HepG2 cells; selective toxicity; protection against hepatocarcinogenesis	(166)
Colon Cancer	HT-29, HCT-15	Gelam, Nenas, Crude honeys	ROS elevation, mitochondrial depolarization, DNA damage, thiol depletion	Dose-dependent anti-proliferative and apoptotic effects; phenolic content correlates with potency	(86, 87, 167)
Prostate Cancer	PC-3	Thyme, Pine, Fir honeys	Cell viability suppression	Only thyme honey showed significant anti-proliferative activity	(165)

Despite promising findings across these varieties, the absence of unified compositional profiling systems makes it difficult to recommend therapeutic doses or compare outcomes across studies. Variability in bioactive components such as MGO, leptosperin, and quercetin underscores the need for standardized metabolomic characterization to identify therapeutic-grade formulations and enable reproducibility in clinical oncology (29, 168).

## 6 Safety, pharmacokinetics, and integration

### 6.1 Safety and risk assessment

Although honey is widely regarded as safe, its therapeutic application requires context-specific evaluation. Contaminants such as heavy metals (arsenic, cadmium, lead), pesticide residues, and hydroxymethylfurfural (HMF) have been detected in some samples, necessitating rigorous quality control and source verification (45). Adverse effects are generally minimal, but certain populations—particularly individuals with type 2 diabetes mellitus (DM2)—may require caution. While some studies suggest honey may serve as a healthier alternative to refined sugars due to its antioxidant and anti-inflammatory properties (169), others report unfavorable metabolic outcomes, including increased LDL cholesterol and reduced adiponectin following daily intake of 50 g natural honey (170). These effects were linked to its high fructose content, with adulteration ruled out. Additional investigations have noted elevations in glycated hemoglobin (HbA1c) at similar doses (171, 172), whereas lower intakes (5–25 g/day) did not significantly affect glycemic parameters (171). Hence, the honey intake should be personalized, particularly among old cancer patients suffering from metabolic comorbidities. Selecting a specific strategy could allow to get the most out of the therapy while at the same time reducing metabolic disturbances. This evaluation of safety does not consider the cases of allergic reactions to plant materials and the risks stemming from the use of impure products, as they categorized under food safety and allergen monitoring practices. Honey usually produces a lower glycemic response than refined sugars. Still, its metabolic effects are dose-dependent, and excessive intake may worsen glycemic control and lipid profiles in patients with diabetes, obesity, or metabolic syndrome. (156). Clinical and preclinical evidence suggests that moderate intakes of Malaysian Tualang honey (5–20 g/day) can be tolerated without adverse metabolic effects, while excessive consumption may exacerbate risks in patients with diabetes or obesity (151, 155). In oncology settings, honey has been used as a supportive adjunct, but patients with metabolic comorbidities represent an at-risk group requiring individualized monitoring (152). These clarifications emphasize that honey should be considered in carefully monitored amounts, with attention to dose ranges and patient risk profiles. In cancer patients with metabolic comorbidities, benefit–risk assessment is critical. While honey provides antioxidant and anti-inflammatory compounds that may support therapy, its glycemic load can exacerbate hyperglycemia, a known risk factor for cancer progression through metabolic reprogramming (Warburg effect). Clinical evidence suggests that small supportive doses (5–20 g/day) are generally tolerated, but in patients with diabetes, obesity, or metabolic syndrome, excessive intake may negate potential antioxidant benefits by worsening glycemic control. Therefore, honey should be considered cautiously in oncology practice, with individualized monitoring of metabolic status to balance therapeutic potential against metabolic risks (151, 152, 156).

### 6.2 Pharmacokinetics and bioavailability

The pharmacokinetic behavior and systemic bioavailability of honey’s bioactive constituents remain poorly defined, posing challenges for its integration into evidence-based oncology. Honey contains sugars, polyphenols, enzymes, and trace compounds. However, the absorption, distribution, metabolism, and excretion (ADME) of these molecules remain poorly defined. (172). Honey contains diverse phenolic and flavonoid compounds whose pharmacokinetics and bioavailability are influenced by both chemical structure and gut microbiota interactions. Studies on pollinator-dependent crops highlight that flavonoid diversity directly affects absorption and metabolic pathways (146). Comparative analyses of conventional and organic honey show differences in phenolic content that translate into variable bioavailability profiles (156). In oncology-related contexts, Malaysian Tualang honey has demonstrated that flavonoids such as quercetin and kaempferol undergo rapid absorption but are extensively metabolized, with gut microbiota playing a role in generating bioactive metabolites (151, 155). Similarly, Sidr honey has been reported to contain phenolic acids whose systemic availability depends on microbial transformation, underscoring the importance of microbiota interactions in therapeutic outcomes (152). These examples clarify ADME processes and highlight how microbiota contribute to the bioavailability of honey’s major bioactive compounds. Emerging data suggest that gastrointestinal pH, enzymatic degradation, and interactions with gut microbiota significantly influence the bioavailability of honey-derived phenolics (63, 171). The absence of standardized analytical techniques for quantifying these metabolites in biological matrices further complicates pharmacokinetic modeling and dose optimization (173). Advancements in metabolomics and nanocarrier technologies may enhance targeted delivery and systemic retention of honey’s therapeutic components. *In vivo* tracer studies are urgently needed to elucidate absorption kinetics and metabolic fate. A further limitation concerns the discrepancy between effective concentrations reported *in vitro* and the actual bioavailability achievable *in vivo*. Many cell culture studies employ phenolic and flavonoid concentrations that exceed those attainable in human plasma following oral ingestion of honey. For example, comparative analyses of conventional and organic honey demonstrate variability in phenolic content, but systemic levels after ingestion remain modest (156). Evidence from Malaysian Tualang honey indicates that compounds such as quercetin and kaempferol undergo extensive metabolism, reducing their circulating concentrations despite high *in vitro* activity (151, 155). Similarly, Sidr honey phenolic acids show therapeutic potential, yet their systemic availability depends on microbial transformation and is lower than *in vitro* dosing (152). These differences highlight the need for cautious interpretation of *in vitro* findings and underscore the importance of pharmacokinetic studies to establish clinically relevant dose ranges.

### 6.3 Integrating honey-based products in clinical oncology

Despite its promising therapeutic profile, integrating honey into mainstream oncology requires a decisive shift from traditional use to evidence-based practice. While preclinical studies have robustly demonstrated honey’s immunomodulatory, anti-inflammatory, and direct anticancer properties, its adoption in clinical protocols remains limited. This gap is primarily due to a scarcity of large-scale randomized controlled trials and persistent regulatory ambiguity (174). Current scientific frameworks increasingly position honey as a supportive agent, particularly for managing treatment side effects like chemotherapy-induced mucositis, accelerating tissue repair, and improving quality-of-life metrics (152). A significant hurdle, however, is its widespread classification as a dietary supplement. This designation typically allows honey to bypass the rigorous safety and efficacy evaluation required for pharmaceutical compounds by agencies like the FDA, thereby complicating its formal approval for clinical use (174).

## 7 Regulatory, ethical, and cultural considerations

The clinical use of honey-based treatments is influenced by a mix of regulatory, ethical, and cultural factors. The international differences in regulatory classifications lead to honey being usually classified as a food or a supplement and, thus, exempt from the standards required for pharmaceuticals such as Good Manufacturing Practices and randomized efficacy trials (175). Such regulatory leniency poses ethical questions about the authenticity of products, safety assurance, and informed consent—especially when honey is marketed with therapeutic claims in oncology contexts (176). Furthermore, cultural beliefs play a major role in this issue, for honey is considered a medicine in traditional systems like Ayurveda and Islamic medicine, which might be at odds with the Western biomedical paradigm (29). Solving these problems will need an interdisciplinary approach that includes global standard unification, clear labeling practices, and culturally appropriate clinical communication. Honey-containing products occupy a complex regulatory space. In the United States, the FDA generally classifies honey as a food product, but honey-based supplements and topical formulations may fall under dietary supplement or drug categories depending on claims made. The European Medicines Agency (EMA) similarly distinguishes between food and medicinal use, requiring evidence for therapeutic claims. Cooperative networks and international organizations have emphasized the need for harmonized standards to ensure safety and fair trade (148, 150). In oncology practice, honey has been used off-label for supportive care, particularly in the management of oral mucositis and wound healing, despite the absence of formal approval for these indications (151, 155). Ethical concerns also arise from marketing practices: the Manuka honey sector in New Zealand illustrates how branding, transparency, and identity crafting can lead to consumer confusion and exaggerated therapeutic claims (153, 154). These examples highlight the importance of regulatory oversight and ethical responsibility in promoting honey-based products for clinical and consumer use.

## 8 The evolution of honey in cancer care

**Figure 2:**
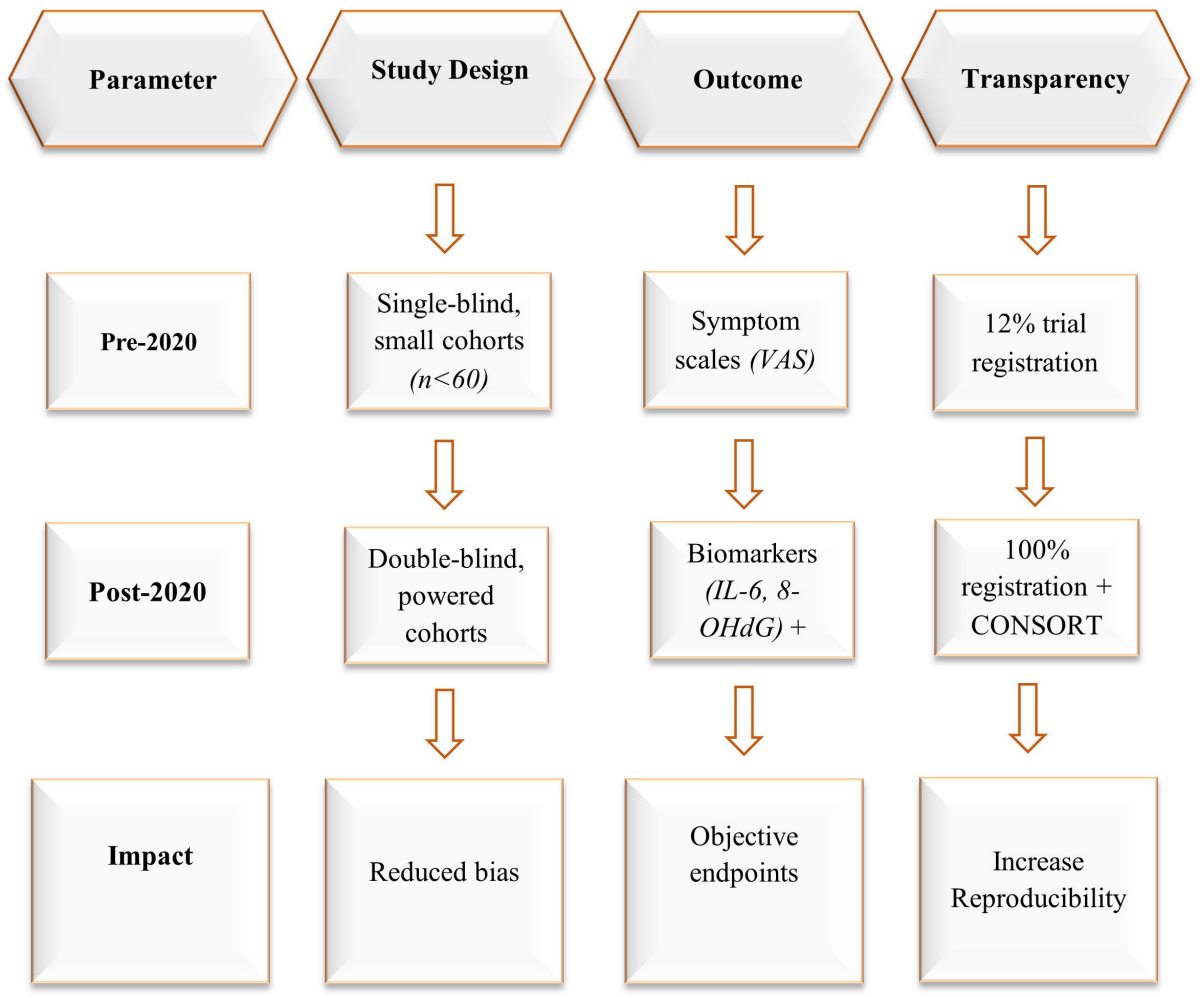
Methodological advances in honey research (159, 177–180).

The therapeutic application of honey in oncology has transitioned from empirical use rooted in traditional medicine to a scientifically investigated adjunct with measurable clinical benefits. Historically, honey was employed for its wound-healing and anti-inflammatory properties, often without mechanistic understanding or standardized formulations (**Figure 2**). Early clinical investigations, particularly those conducted prior to 2020, were limited in scope—characterized by small sample sizes, lack of blinding, and endpoints focused primarily on symptomatic relief (159, 177–180). In contrast, post-2020 research reflects a paradigm shift toward precision medicine (**Figure 2**). Recent studies incorporate biomarker-based endpoints, rigorous trial designs, and chemically characterized honey preparations. These developments have made it possible to conduct with greater certainty evaluations of honey’s role in cancer-related consequences, which consist of the suppression of inflammatory cytokines, the strengthening of antioxidant defenses, and the reduction of the toxicities induced by treatments (159, 177–180). The combination of molecular profiling and standardized dosing protocols signifies a major change in the evidence base, which now regards honey as a candidate for structured inclusion in integrative oncology.

## 9 Challenges and future directions

The therapeutic promise of honey in oncology is supported by a robust foundation of preclinical evidence, yet its clinical translation remains hindered by several unresolved challenges. Although numerous *in vitro* and animal studies have demonstrated honey’s ability to inhibit tumor proliferation, induce apoptosis, suppress inflammatory signaling, and impair metastatic progression—effects largely attributed to its rich phenolic and flavonoid content—human trials remain limited in scale and methodological rigor (29, 159, 181). A primary obstacle lies in the intrinsic variability of honey’s composition. Factors such as floral origin, geographical location, seasonal variation, and post-harvest processing significantly influence its biochemical profile, resulting in inconsistent concentrations of key bioactives like methylglyoxal, leptosperin, and quercetin (29). This heterogeneity complicates reproducibility across studies and impedes the development of standardized dosing protocols. Equally problematic is the lack of comprehensive pharmacokinetic data. A critical challenge in the clinical translation of honey is the incomplete understanding of its pharmacokinetics—specifically, how its active components are absorbed, distributed, metabolized, and excreted (ADME) in the body (182). This knowledge gap makes it difficult to determine effective clinical dosages based on promising *in vitro* results, leaving therapeutic optimization largely speculative. To bridge this divide, a concerted multidisciplinary research strategy is essential. Rigorous clinical trials with biomarker-driven endpoints are essential to validate honey’s efficacy in cancer prevention and treatment. Molecular profiling of honey varieties using metabolomics and chemometric tools can facilitate the identification of therapeutic-grade formulations. Integration with nanocarrier systems—such as liposomes, dendrimers, and polymeric nanoparticles—may enhance targeted delivery, improve stability, and increase systemic retention of bioactive compounds. Furthermore, systems biology approaches, including transcriptomic and proteomic analyses, can elucidate honey’s interactions with tumor microenvironments and immune networks, offering mechanistic insights into its therapeutic actions. Collectively, these efforts will be pivotal in transitioning honey from a traditional remedy to a scientifically validated adjunct in precision oncology, capable of complementing conventional therapies and improving patient outcomes (29, 119, 182).

## 10 Strengths and limitations

The present study has specific importance by integrating sustainability perspectives with clinical oncology, drawing on diverse sources including ecological, socioeconomic, and therapeutic evidence (145, 146, 148). The use of vetted references across different honey types (Tualang, Manuka, Sidr) provides a broad comparative framework (151, 152, 154). Furthermore, the study highlights regulatory and ethical considerations, linking scientific findings to policy and practice (149, 150).

However, limitations must be acknowledged. The analysis relies primarily on published literature rather than new clinical trials, which restricts the ability to define precise dose ranges and pharmacokinetic parameters. While examples of metabolic and microbiota interactions are provided, more detailed ADME data from controlled oncology studies are needed (155, 156). Additionally, the scope of regulatory review is limited to selected authorities, and marketing claims were illustrated mainly through the Manuka honey sector, which may not generalize globally. These limitations highlight areas for future research, including clinical dose-response studies, broader regulatory mapping, and expanded evaluation of honey’s therapeutic applications in oncology.

## 11 Conclusions and future directions

Honey demonstrates multifaceted anticancer potential, exerting effects that align with several hallmarks of cancer, including apoptosis induction, inhibition of proliferation, suppression of metastasis, and mitigation of treatment-induced toxicities. These activities are mediated by its diverse bioactive constituents such as polyphenols, flavonoids, and methylglyoxal, which influence pathways ranging from caspase activation and cell cycle arrest to NF-κB inhibition and antioxidant enzyme upregulation. Despite this promise, the current evidence base is dominated by preclinical studies, with only a limited number of small clinical trials providing supportive data. As such, honey should presently be regarded as a supportive adjunct rather than a validated therapeutic agent in oncology.

Future research must prioritize large-scale randomized controlled trials with biomarker-driven endpoints to establish clinical efficacy. Standardized compositional profiling beyond the UMF™ grading system is urgently needed, incorporating metabolomic markers directly linked to anticancer activity to overcome variability between honey types such as Manuka, Tualang, and Sidr. Pharmacokinetic studies are also essential to define the absorption, distribution, metabolism, and excretion (ADME) profiles of honey’s bioactive constituents, which remain poorly characterized. Advances in nanotechnology, including honey-loaded nanoparticles and liposomal formulations, offer promising strategies to improve bioavailability and targeted delivery. Finally, systems biology and omics-based approaches, particularly proteomics and transcriptomics, can provide comprehensive insights into honey’s interactions with tumor biology and host immunity.

Taken together, this balanced perspective highlights both the opportunities and limitations of honey in cancer care. While its traditional use and emerging scientific validation underscore its potential, rigorous clinical and translational research will be pivotal in determining whether honey can transition from a natural remedy to a scientifically established adjunct in precision oncology.
